# Insight in the diagnosis and treatment of coeliac disease in general practice: A survey and case vignette study among 106 general practitioners

**DOI:** 10.1080/13814788.2021.1985455

**Published:** 2021-11-08

**Authors:** Maxine D. Rouvroye, Pauline Slottje, Tom van Gils, Chris J. Mulder, Jean W. Muris, Dick Walstock, Marcel Reinders, Gerd Bouma

**Affiliations:** a Department of Gastroenterology and Hepatology, Amsterdam Gastroenterology & Metabolism, Amsterdam UMC, Vrije Universiteit Amsterdam, Amsterdam, the Netherlands; b Department of General Practice and Elderly Care Medicine, Amsterdam UMC, Vrije Universiteit Amsterdam, Amsterdam, the Netherlands; c Department of General Practice and Elderly Care Medicine, Amsterdam UMC, Vrije Universiteit Amsterdam, Academic Network of General Practice, Amsterdam, the Netherlands; d Department of Family Medicine, Care and Public Health Research Institute CAPHRI, Maastricht University, Maastricht, the Netherlands

**Keywords:** Coeliac disease, gluten, diet, general practice, questionnaires and surveys

## Abstract

**Background:**

Coeliac disease (CD) is a highly prevalent (∼1%) disease that allegedly remains undiagnosed in over 80% of the cases because of atypical symptoms or silent disease. Currently, it is unknown how GPs deal with (suspected) CD.

**Objectives:**

This study aimed to better understand the diagnostic approach and the clinical reasoning process of GPs concerning CD and concurrently address diagnostic pitfalls.

**Methods:**

A questionnaire with case vignettes to assess the knowledge, diagnostic reasoning pattern and practice for CD by GPs was developed. It was sent through academic GP research networks (encompassing over 1500 GPs) in two large cities and to smaller practices in rural areas. The questionnaire was composed of seven background questions, 13 questions related to four case vignettes and six additional CD-related questions

**Results:**

Responses were received from 106 GPs. Knowledge on risk factors for CD and appropriate testing of at-risk populations was limited. Twenty-two percent would diagnose CD in adults exclusively based on serology, without histopathological confirmation. In total, 99% would refer a newly diagnosed patient to a dietitian to initiate a gluten-free diet (GFD). In the absence of symptoms, only 33% would initiate a GFD.

**Conclusion:**

The results of this study have given us insight into the diagnostic process of GPs encountering patient with gluten-related complaints. Multiple serology test is available and used, while a positive serology test is not always followed up by a gastroduodenal biopsy to confirm the diagnosis. Most GPs would refer a symptomatic CD patient to a dietician for a GFD.

KEY MESSAGES

- Classic symptoms mainly prompt testing for CD
- Many risk factors of CD are not recognised as such
- There is heterogeneity in types and timing of testing

## Introduction

Coeliac disease (CD) is a chronic, immune-mediated disease triggered by dietary gluten in the small intestine in genetically susceptible individuals [1]. The prevalence of CD is 0.8% in Europe [1,2]. Diagnosis in adults relies on serological screening (tissue-Transglutaminase IgA antibodies (tTGA)), and if these are abnormal, followed by histopathological examination of duodenal biopsies [3]. The majority of the paediatric population can be diagnosed by serological testing only, confirmed by a paediatrician [4]. Treatment consists of a strict life-long gluten-free diet (GFD) [1]. Education, assessment of nutritional status and follow-up, preferably by a trained dietitian, are key to strict adherence and an optimal health status [5]. Despite the high sensitivity and specificity of diagnostic tests, CD is still underdiagnosed [6,7]. This may partly be explained because the classical symptoms of bloating, diarrhoea, and abdominal pain are generally not found in adults [8]. First-degree relatives have a 5–10% risk of CD, while the prevalence of CD among second-degree relatives was 1:39 (2.5%) [9]. Apart from classic symptoms, (otherwise idiopathic) iron deficiency anaemia, Cryptogenic hypertransaminasaemia, osteoporosis, skeletal fractures, psoriasis, recurrent aphthous ulcerations, recurrent miscarriage, diabetes type 1, thyroid disease, Turner syndrome, Down’s syndrome should also arouse suspicion of CD and prompt serological testing [10–14]. The rise in self-diagnosed gluten sensitivity and self-initiation of a GFD in western societies also makes it difficult to separate the wheat from the chaff as gluten containing diet is essential for diagnosing CD [15]. A qualitative study performed by our group featuring in-depth interviews on awareness, diagnostics and management of CD among seven GPs exhibited very diverse diagnostic and treatment strategies between GPs [16]. A discordance in views of age groups at-risk and overall at-risk groups was described, as well as different testing methods for CD or follow-up methods. To better understand why CD is underdiagnosed we have now performed a follow-up study to understand how GPs recognise, assess and manage (suspected) CD. This study aimed to assess the knowledge on CD amongst GPs and their diagnostic thinking process regarding (suspected) CD, address diagnostic pitfalls and concurrently raise (self) awareness among GPs regarding CD.

## Methods

### Study design

We developed an online questionnaire for GPs concerning their diagnostic reasoning, practice and their clinical management approach regarding CD. The questionnaire covered background characteristics of respondents, six questions on general CD knowledge and 13 questions, related to four case vignettes (Table 1). All case-related questions were multiple choice, including one option with free text space for other answers. Two GPs, a gastroenterologist and an epidemiologist pilot-tested the questions and items were refined based on their extensive feedback. The questionnaire was written in Dutch (see Supplementary data  for an English version).

**Table 1.** Case descriptions.

*Case 1*	A 51-year-old menopausal woman with a history of refractory iron deficiency anaemia, miscarriage and thyroid disease, presents with fatigue, weight loss, bloating and constipation. Respondents were asked to form a top five differential diagnoses from a list of 19 choices and the opportunity was given to add a diagnosis in a blank space. In the following question, participants were asked to select the tests they would request for the diagnostic workup from a list similar to those used in clinical practice. Laboratory test forms from three different regions served as an example. Subsequently, the case description continued that the tTGA titre turned out to be 120U/ ml (reference valu*e* < 7U/ ml). With this knowledge, the respondents were asked questions about completing the diagnosis, treatment and follow-up. Seven general questions to test the knowledge on CD prevalence in de general practice, available diagnostic tests were positioned halfway the first case.
*Case 2*	A 25-year-old student experiencing abdominal pain, bloating and changing bowel habits for seven years, and therefore self-initiated a gluten-free diet a year ago with gradual relief of her symptoms. The accompanying questions concerned diagnostic options for CD in patients currently on a gluten-free diet.
*Case 3*	A 2.5-year-old toddler with failure to thrive, extensive crying, and a mother with CD. The questions were designed to test the knowledge on paediatric CD and screening in first-degree relatives.
*Case 4*	A 28-year-old female that was diagnosed with CD *via* screening. Since she does not experience any symptoms, the question has arisen whether a gluten-free diet is required in her situation.

### Selection of study subjects

General practitioners from three urban areas in the west and the south of the Netherlands were approached; this concerned the Network of General Practice of the Amsterdam UMC, location VU medical centre, the general practice cooperation Zuid-Kennemerland, and the Research Network Family Medicine Maastricht. We also approached GPs from more rural areas in the vicinity of Abcoude, Leiden and Arnhem. General practitioners were invited to fill out the questionnaire *via* email, and a message posted on their organisation’s intranet. The questionnaire was available for ten months, from January 2018 onwards. Due to privacy regulations, we could not keep track of the exact amount of invitees and we do not know precisely how many GPs received an invitation. However, the GP networks we approached roughly encompass about 1700 GPs. All invitees were informed about the gastrointestinal diagnostics topic, yet blinded to the specific CD subject of the questionnaire; first to decrease the chance of inclusion bias (e.g. those with CD interest) and second to properly uncover the differential diagnostic thinking process of GPs regarding suspected CD. Searching for answers while filling in the questionnaire was discouraged in the invitation. No incentive was offered to participants and no reminders were sent.

#### Data collection and analysis

Collector 2015.Q2, a programme used to build questionnaires and approved by the Amsterdam UMC, was used to create the questionnaire, distribute it (i.e. providing a non-personalised link), and merge and extract all answers. Questions were presented to participants, one question per window, without the option to return to previous questions to change the respective answers. Descriptive analysis was performed using SPSS22.0. Continuous data were compared using Mann–Whitney U-test, whereas categorical data were compared using Chi-square test. Questionnaires that were not completed were excluded from the analysis.

#### Ethics

This study was approved by the local Medical Ethics Review Committee of the VU University Medical Centre (number 2016-499). Informed consent was integrated into the online questionnaire, which could be completed anonymously. The study protocol conforms to the ethical guidelines of the 1975 Declaration of Helsinki as reflected in a priori approval by the institution's human research committee.

## Results

Of all GPs to whom we sent an email, 164 GPs opened the link to the online questionnaire and 106 (64.6%) of them completed it. We did not detect clinically relevant differences in the assessment of the cases and additional questions between GPs regarding age, gender, years of experience or number of inhabitants per city/village they worked in.

### General knowledge on coeliac disease

The participants estimated a median prevalence of CD in the general population of 2.0% [95% CI 2.0–4.0], and deemed that 27.5% [95% 20.0–36,5] of these CD patients are currently diagnosed. Almost sixty percent (58.5%) of all participants voted for the most accurate incidence CD curve with a high peak around the age of two and a lower curve from 18-year old to an elderly age (Supplementary data).

When asked about the test characteristics of the tTGA test, 40% (*N* = 43) considered sensitivity and specificity of both 70% most applicable, 33% (*N* = 36) chose the answer closest to reality with a sensitivity and specificity of both 95%. Twenty-two percent (*N* = 24) of the respondents deemed a positive tTGA test is sufficient to establish a CD diagnosis in adults. Solely based on a positive tTGA test 48% (*N* = 52) would recommend a GFD. Of these, 46% (*N* = 24) would refer patients to a dietitian to initiate a GFD based on the positive tTGA test and 40% would refer the patient to a gastroenterologist.

**Table 2.** Diseases or symptoms prompting participants to test for coeliac disease.

Possibilities	Participants that would test for CD (%)	WGO Global Guidelines	ESsCD
Persisting fatigue	69%	X	X
Irritable bowel syndrome (Rome criteria)	77%	X	X
Idiopathic ataxia	7%	X	X
Oral aphthous ulcers	20%		X
Heart failure*	2%		
Hypothyroid disease	19%	X	X
1st degree family member with CD	88%	X	X
Weight loss	90%	X	X
Psoriasis	6%		X
Osteoporosis/Low bone density	36%	X	X
Down's syndrome	15%	X	X
Asthma*	5%		
Type-1 diabetes	17%	X	X
Type-2 diabetes*	4%		
Idiopathic subfertility	15%	X	X
Idiopathic neuropathy	15%		X
Dermatitis herpetiformis	16%	X	X
Enamel defects	7%		X
Recurrent otitis*	2%		
Other: anaemia, B12-deficiency, abdominal pain, diarrhoea, diarrhoea after eating bread, duodenal diarrhoea, obstipation	10%	X	

The percentage of participants that would opt for a coeliac disease test in case of the following symptoms, disorders or a family history of CD. A few hoax options were given and indicated with an asterisk. The following columns indicate whether the World Gastroenterology Organisation Global Guidelines and the European society for the study of coeliac disease advise serological testing for CD (marked with an X). The last row is an accumulation of options that were added by participants themselves (in total 10%). CD: coeliac disease; WGO: World Gastroenterology Organisation; ESsCD: European Society for the study of Coeliac Disease.

Most GPs would consider testing for CD in case of more conventional symptoms like gastrointestinal symptoms, weight loss and fatigue. Less apparent symptoms or risk factors (e.g. osteoporosis, diabetes type I, enamel defects) were less frequently a reason for testing for CD (Table 2).

Thirty-five percent of the GPs considered CD in their top five differential diagnoses in case 1 describing a middle-aged woman with weight loss and abdominal complaints (Supplementary data), right behind irritable bowel syndrome, iron deficiency anaemia, gastrointestinal malignancy and thyroid disease (from multiple choice of 19 options and an option to add another diagnosis. Diagnostic tests for CD were the eleventh most commonly requested test in this case vignette (Table 3).

**Table 3.** Laboratory tests requested in case 1.

Type of laboratory test requested	Times requested [*N* (%)]
Thyroid-stimulating hormone	124 (92%)
Haemoglobin	122 (90%)
Erythrocyte sedimentation rate	99 (73%)
Glucose	73 (54%)
Mean corpuscular volume	71 (53%)
Alanine aminotransferase	66 (49%)
Ferritin	65 (48%)
Leucocytes	57 (42%)
Creatinine	52 (39%)
Vitamin B12	52 (39%)
Free T4	46 (34%)
Coeliac disease diagnostics	*43 (32%)*

In this case, the tTGA test was positive; 78% did not regard a tTGA test to be sufficient for diagnosis CD, 22% (*N* = 24) of the respondents deemed a positive tTGA test to be sufficient to establish a CD diagnosis in adults. Regardless, 48% (*N* = 52) would recommend a GFD solely based on a positive tTGA test, 46% (*N* = 24) would refer patients to a dietitian for the initiation of a GFD. Of these GPs, 19 would adopt a two-track policy by also referring the patient to a gastroenterologist.

When participants were asked what type of additional examinations should be performed by a gastroenterologist after referral, all GPs chose an endoscopic examination; 77% (*N* = 83) chose a gastroduodenoscopy with biopsies, 3% (*N* = 3) chose a colonoscopy without biopsies, 26% (*N* = 28) chose a colonoscopy with biopsies, one participant chose a gastroduodenoscopy without biopsies and none chose radiological exams (abdominal MRI or CT). A biopsy result of total villous atrophy, crypt hyperplasia and increased intra-epithelial lymphocytes convinced 90% of the participants that the diagnosis of CD can be established. After confirmation of the diagnosis, 99% of the participants would refer the patient to a dietitian instead of recommending a self-instituted GFD.

In total, 79% opted for active follow-up (either every 3 or 6 months) with blood testing, 19% opted for a passive follow up plan in which the patient has to seek help actively in case of symptoms, and 2% stated that no follow-up was needed.

The second case dealt with the dilemma of diagnostic testing in a patient on a self-initiated strict GFD. One percent would perform HLA-DQ typing and 39% would opt for a gluten challenge (reintroduction of gluten in the diet and a tTGA test after a period of gluten intake). The median time suggested by participants for the gluten challenge was 6.1 ± SD4.5 weeks. Twelve percent would refer the patient to a gastroenterologist. Remarkably, 33% would opt for a tTGA test or a gastroduodenoscopy, while neither is appropriate in a patient following a GFD for a more extended period. Four percent would choose not to perform any tests since the patient does not experience symptoms.

Based on the provided information in case 3 regarding a toddler, 90% would refer the child to the paediatrician, 3% to the gastroenterologist, 2% to a dietitian, 2% would request a gastroduodenoscopy with biopsies directly and 3% would start a GFD purely based on the complaints and the tTGA titre. Fifty-nine percent would perform screening in (symptom-free) siblings (18% HLA-DQ typing, 78% tTGA test, 3.6% referral to a paediatrician), 41% would not test the siblings.

Thirty-one percent of the participants would start a GFD in a symptom-free patient, 7% would seek advice from a gastroenterologist, 24% would not advise a GFD and would rather have the patient return in case of symptoms, 21% would advise to limit the gluten intake but not fully refrain from it. Additionally, 36% of all GPs would recommend annual blood tests regardless of the beforementioned treatment strategies.

## Discussion

### Main findings

In this questionnaire-based study, we identified gaps in knowledge, diagnostic reasoning and management of CD by GPs. Although most GPs answered questions correctly, there was a heterogeneity in recognising risk factors, the use of diagnostic tests and the appropriate timing for testing. The intention to test for CD is more frequent in a patient with classic CD symptoms and less apparent in case of certain risk factors (e.g. Down’s syndrome, osteoporosis). A third of the GPs would test for antibodies or request a duodenoscopy while a patient has already eliminated gluten from the diet, indicating a lack of knowledge of the underlying pathophysiology of CD.

### Strengths and limitations

This study was the first to investigate how GPs deal with CD-related complaints, diagnosis, treatment and follow up using a questionnaire built around 4 case vignettes and it has brought forth valuable information for reflection. Participants did not know beforehand that the subject of the questionnaire was CD, limiting the chance of selection bias. The study was performed in the Netherlands, and most participants were approached through academic networks. Therefore, results might not directly apply in other European countries.

### Interpretation of the study results in relation to existing literature

Only 35% of the participants considered CD in their top five differential diagnoses in case 1 and 32% of all participants (*N* = 43) ordered CD diagnostic tests (among other diagnostic tests). Following both global and European gastroenterology guidelines, six CD-related signs and symptoms that individually justify serological testing for CD were reported in description of the first case [17,18]. Active case finding in at-risk groups, like first-degree relatives (case 3) and symptomatic cases (case 1 and 3) is advised since the diagnostic yield is increased [4,17]. However, the diagnostic yield for CD in Dutch patients visiting the GP because of a suspicion of CD was found to be only 1.6% [19]. If patients are already on a strict GFD like case 2, diagnosis is more complicated.

In some cases, serology and biopsy are still positive within three months of initiating a GFD. In other cases, an HLA-DQ typing can be performed to rule out CD. However, diagnosis cannot be based solely on HLA-DQ2/8 positivity since up to 40% of the Caucasian population also carries one of these haplotypes [1]. A gluten challenge is required to diagnose CD in such cases, although newer methods that are less burdensome for the patient are on the horizon [20,21].

The sensitivity and specificity of tTGA are 93.0 and 96.5, respectively [22]. For children, the accuracy is even higher. This accuracy was underestimated by 67% of the participants. Nevertheless, 48% would initiate a GFD in adults with a high tTGA titre without further examination. According to international guidelines, a histopathological examination of duodenal biopsies is still required in adults to establish a CD diagnosis [17]. Ninety-nine percent of the participants would refer the patient (case 1) to a dietician to initiate a GFD. The help of a registered dietitian is imperative in educating patients and preventing additional healthcare costs (5) and strict adherence is associated with a better quality of life [23,24].

Adults with screen-detected CD may also benefit from a GFD, regardless of symptoms [25]. Nevertheless, only 31% of GPs would recommend a GFD in an asymptomatic patient diagnosed through case finding (case 4). Vilppula *et al.*, demonstrated anaemia (13%), osteoporosis and/or osteopenia (62%), and a history of low energy fractures (23% vs 4% in background population) in screen-detected adults [25]. In these patients, Vitamin B12, Vitamin D and folic acid levels increased significantly after initiation of a GFD, and alleviation of gastrointestinal symptoms was observed, even though patients did not report having these symptoms at first (25). The decision to maintain a gluten-containing diet should, therefore, be well-considered. Little is known about the follow-up of CD patients. In a questionnaire among CD patients in the United Kingdom, a follow-up rate of 62% was observed after a median follow duration of 5.4 years, 8% in primary care and 92% in hospital [26]. Dutch and European guidelines propose that this task lies with gastroenterologists and paediatricians [17,27]. However, it is still debateable whether the general practice might not be a better place for CD follow up as it is more cost-effective and preferred by the patient [26]. Textbook 1 summarises the case vignette’s learning points.

Textbox 1. The case vignette’s learning points

- The prevalence of biopsy-proven CD worldwide is around 0.7%, although prevalences vary greatly among populations with ∼0.2-0.35% in the Netherlands and Germany. Coeliac disease should also be considered in adults.
- The golden standard in the diagnosis of CD is based on serological testing of antibodies against tTGA followed by immunohistochemical examination of a duodenal biopsy. In children, a biopsy is not always needed.
- If a patient has been adhering to a GFD for a longer time negative HLA-DQ2/8 genotyping result obviates the need for further workup. If positive, a gluten challenge is needed before testing for tTGA. A period of 6–8 weeks of eating 10g gluten a day is recommended, although some studies indicate a shorter period is sufficient as well.
- All children with a positive tTGA should be referred to a paediatrician/paediatric gastroenterologist for further workup. It is advised to test first degree relatives for CD; a negative HLA-DQ2/8 test can exclude the diagnosis of CD and the possibility of developing CD later in life.
- A beneficial effect of a GFD was also observed in asymptomatic patients regarding energy level, the risk of osteoporosis and vitamin deficiencies. However, the long-term outcome of untreated CD is still relatively unclear and the quality of life while adhering to a GFD should be taken into consideration while deciding on a GFD or follow-up method.

### Implications for clinical practice and future research

The current study could serve as a reflection tool and an opportunity to refresh the knowledge on CD. To provide relevant recommendations for GPs, evidence should essentially be extracted from general practice-based studies [28]. In the Netherlands, no specific CD guideline for GPs exists. The GP guidelines on food intolerance and irritable bowel syndrome provide guidance on which serological tests can be used to exclude CD in the diagnostic workup [27,29]. In the United Kingdom, a CD guideline for GPs is provided and updated by the National Institute for Health and Care Excellence [30]. A similar European guideline might increase the uniformity in diagnostic behaviour and the diagnostic yield of CD by GPs. Based on our results, emphasis should be placed on the recognition of risk factors for CD and the diagnostic process.

## Conclusion

In this questionnaire-based study, we identified gaps in knowledge, diagnostic reasoning and management of CD by GPs.

The study results provided insight into the clinical reasoning process of GPs regarding patients with gluten-related complaints. The encountered diagnostic pitfalls are characterised by the availability and alternate use of multiple serological tests and the frequent absence of a gastroduodenal biopsy to confirm the diagnosis of CD in patients with a positive serological test. Most participants would refer a patient with CD and complaints to a dietician to initiate a GFD. Only one third of participants would advise a GFD in asymptomatic patients.

## Supplementary Material

Supplementary Data
