# Supplementary material for: Insight in the diagnosis and treatment of coeliac disease in general practice: A survey and case vignette study among 106 general practitioners
Source: Eur J Gen Pract. 2021 Nov 8;27(1):313–9. doi: 10.1080/13814788.2021.1985455 (PMC8583831; doi:10.1080/13814788.2021.1985455)
Supplement: Supplementary Data [file IGEN_A_1985455_SM6529.docx]

Supplementary data 1: the questionnaire translated into English

**Demography**:

1. Will you give your permission to use anonymised data filled in by you in the following questionnaire for research purposes?
   - Yes
   - No
2. What is your gender?

- Female
- Male

1. What is your current age (in years):

... years

1. Years of experience as a general practitioner (residency not included):

… years

1. How many inhabitants are registered in the city/village where your practice is located?

- >250.000
- 50.000-250.000
- 10.000-50.000
- <10.000

1. Do you have a registered dietitian working in your practice or do you have a partnership with a dietitian with whom you regularly treat patients?

- Yes
- No

1. Estimate the total amount of patients in your practice (treated by you)

… patients

1. Previous experience as an intern or resident (multiple options):

- Paediatrics
- Internal medicine
- Gastroenterology
- Surgery
  1. Other: …
- Specialist/consultant (other than GP)

## Case 1

Female patient, 51-years-old, elementary school teacher

Medical history

1987 Recurrent iron deficiency anaemia for which she received iron tablets

1994 Miscarriage

2005 Subclinical hypothyroid disease

Reason for consultation: Fatigue

Anamnesis:

She has been fatigued for several months and has lost over 8 pounds in twelve months without any changes in her diet or more exercise. Gastrointestinal complaints for two years, abdominal bloating at the end of the day, constipation, no diarrhoea, no blood loss or mucus, no family history of intestinal cancer. Gynaecological: post-menopausal at the age of 45-years-old.

1. **What is your differential diagnosis (please choose 5 options from the list below or add a new option)**

Anemia

Iron deficiency

Vitamin B12 deficiency

Folic acid deficiency

Vitamin D deficiency

Hypothyroid disease/ Hashimoto’s disease

Diabetes

Menopausal symptoms

Burn out

Depression

Chronic infection

Infectious mononucleosis

Gastro-intestinal infection (e.i. Dientamoeba fragilis)

Gastritis or reflux esophagitis

Inflammatory bowel disease (Crohn’s disease or ulcerative colitis)

Irritable bowel syndrome

Coeliac disease

Gastro-intestinal malignancy

(Congestive) heart failure

Other: …

1. **You decide to perform some laboratory tests. Which tests would you request in this stage of the diagnostic process? (multiple options are allowed and/or add a new option)**

| **Test** |  | **Test** |  | **Test** |  |
| --- | --- | --- | --- | --- | --- |
| Hemoglobin |  | Creatinine |  | HLA-B27 |  |
| Hematocrit |  | Uric acid |  | Antinuclear antibody |  |
| Mean corpuscular body |  | Sodium |  | Anti-neutrophil cytoplasmic antibodies |  |
| Leucocyte count |  | Potassium |  | Anti-mitochondrial antibodies |  |
| Thrombocyte count |  | Calcium |  | Coeliac disease test package |  |
| C-reactive protein |  | Magnesium |  | Anti-gliadin antibodies |  |
| Erythrocyte sedimentation rate |  | Ferritin |  | Deamidated anti-gliadin antibodies |  |
| Glucose |  | Reticulocyte count |  | Anti-endomysial antibodies |  |
| HbA1c |  | Iron |  | tissue transglutaminase antibodies |  |
| Thyroid-stimulating hormone |  | Folic acid |  | HLA-DQ2.5 / 8 |  |
| Free T4 |  | Vitamin B12 |  | **Stool samples** |  |
| Alanine transaminase |  | Parathyroid hormone |  | iFOBT |  |
| aspartate transaminase |  | Vitamin D |  | Parasites |  |
| γ-glutamyl transpeptidase |  | Epstein Barr-virus |  | Faecal calprotectin |  |
| Alkaline phosphatase |  | Borrelia burgdorferi |  | **Urine** |  |
| Lactate dehydrogenase |  | CA 19-9 |  | Natrium, creatinine |  |
| Bilirubin |  | Carcinoembryonic antigen |  | Dipstick |  |

*In this case, coeliac disease might be at the top of your differential diagnosis list. The following questions are about coeliac disease. After this, we will return to the case with our (blood) test results.*

## General questions on coeliac disease

1. **Estimate the prevalence (%) of coeliac disease in the general population**

...%

1. **Estimate the percentage of the general population in The Netherlands that is diagnosed with coeliac disease**

...%

*Classic symptoms and extra-intestinal symptoms or disorders related to or caused by coeliac disease*

*Classic symptoms of coeliac disease; diarrhoea, weight loss, abdominal bloating are far less common than expected. This makes it difficult to diagnose the disease. Patients often portray extra-intestinal symptoms. Furthermore, coeliac disease may present in combination with other disorders (mainly on a genetic basis)*

1. **Which of the following symptoms or disorders should prompt testing for coeliac disease? (multiple options are allowed and/or add a new option)**

| Chronic fatigue |  | Osteoporosis |  |
| --- | --- | --- | --- |
| Irritable bowel syndrome |  | Down’s syndrome |  |
| Idiopathic ataxia |  | Asthma |  |
| Aphthous stomatitis |  | Diabetes type I |  |
| Heart failure |  | Idiopathic subfertility |  |
| Hypothyroid disease/Hashimoto |  | Dermatitis herpetiformis |  |
| 1^st^-grade family member with coeliac disease |  | Idiopathic peripheral neuropathy |  |
| Weight loss |  | Enamel defects |  |
| Psoriasis |  | Recurrent ear infections |  |
|  |  | Other: …. |  |

1. **Which of the following curves is the most representative depiction of incidence of the general population with regard to age at diagnosis.**

**
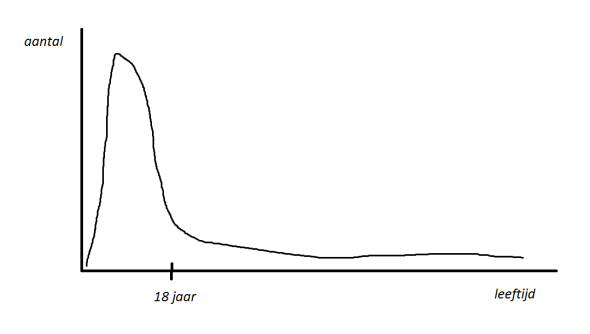
** **
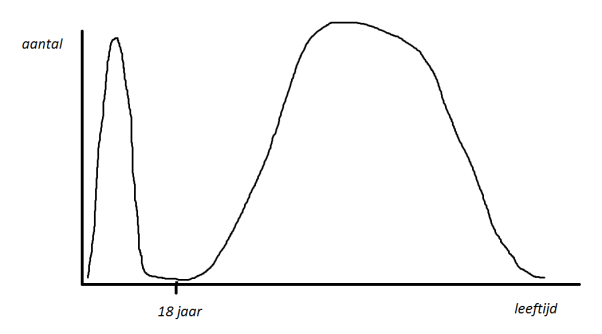
**


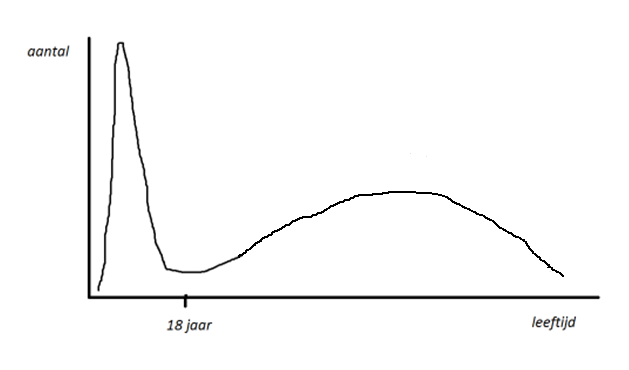

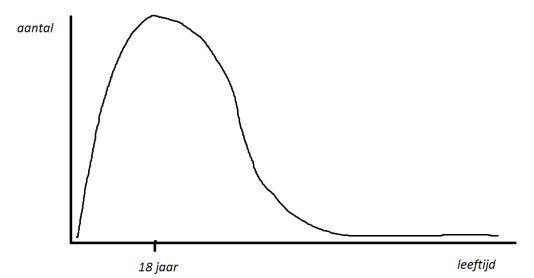


*Suppose you have a patient with a strong suspicion of coeliac disease and you order a serological blood test for coeliac disease. The gold standard in diagnosis is a serological anti-tissue transglutaminase IgA antibody test (anti-tTGA IgA).*

1. **Which of the following descriptions of the testing capabilities of an anti-tTGA IgA is closest to reality?**

- Sensitivity 25%, Specificity 95%
- Sensitivity 70%, Specificity 25%
- Sensitivity 70%, Specificity 70%
- Sensitivity 95%, Specificity 25%
- Sensitivity 95%, Specificity 95%

1. **Anti-tissue transglutaminase IgA antibody point-of-care tests and at-home tests are available online and at drugstores (generally cheaper than ELISA tests). Do you think these tests have similar, better or worse testing capabilities?**

- Similar
- Better
- Worse

*We will now proceed with the first case.*

*Please review the following test results:*

| **Test** | **Result (normal value)** |
| --- | --- |
| anti-tTGA IgA | 120 U/ml (< 7 U/ mL) |
| IgA deficiency | No |

1. **Do you have enough information to establish a diagnosis?**

- Yes
- No

1. **What would be your next step in the diagnostic process (multiple options are allowed and/or add a new option)**

- The initiation of a gluten-free diet
- Additional blood tests: …
- Order a gastroduodenoscopy
- Order a colonoscopy
- Refer to a dietitian for advice
- Refer to specialist
- Other: …

1. **You have referred your patient to a gastroenterologist to complete the diagnostic workup. What examinations or tests should the gastroenterologist perform according to you? (multiple options are allowed and/or add a new option)**

- Gastroduodenoscopy with duodenal biopsies
- Gastroduodenoscopy without duodenal biopsies
- Coloscopy with colonic biopsies
- Coloscopy without colonic biopsies
- Abdominal CT
- Abdominal MRI

*A gastroduodenoscopy was performed and biopsies were taken. During histopathological examination of the duodenal biopsies a total villous atrophy, crypt hyperplasia and intraepithelial lymphocytes were observed.*

1. **Based on these findings, can you now establish the diagnosis of coeliac disease?**

- Yes
- No
  - Why not …

1. **How would you support your patient with the treatment?**

- I will provide the patient with the necessary advice so she can initiate the gluten-free diet independently
- I will refer her to a (specialised) dietitian
  - Provide advise and allow the patient to start a gluten-free diet independently
  - Optional blanc space to clarify your answer

1. **Would you advise a follow-up to monitor the disease in the patient?**

- There is no need for follow up
- Passive follow-up: let the patient return in case of symptoms or complaints
- Active follow-up
- I would have a different plan, I would …

_ The end of case 1 _

## Case 2 to 4

## Case 2

A twenty-five-year-old female hotel management student

Reason for consultation: Suspicion of coeliac disease

Anamnesis:

She has been suffering from abdominal pain and bloating and changing bowel habits. She is exasperated after night shift and exhausted for a week. After reading a newspaper article on ‘gluten allergy,’ in which the same symptoms she suffered from were described, she has banned gluten from her diet. She has kept to a strict gluten-free diet for a year now and all her complaints have disappeared gradually. However, she does want to know whether she has coeliac disease.

1. **How would you approach this case, and what would be your first step in the diagnostic process (**multiple options are allowed and/or add a new option**)?**

- I would not do anything since all her complaints have disappeared
- Re-introduction of gluten in the diet in preparation of a gluten challenge
- Referral to a gastroenterologist
- Anti-tTGA IgA serological test
- HLA-DQ2.5 / DQ8 typing
- Gastroduodenoscopy with duodenal biopsies
- Other: …

*Individuals that carry the immunomodulating HLA-DQ type 2 or 8 are genetically predisposed to coeliac disease. In total, 95% of the coeliac disease patients carry HLA-DQ2.5, and 5% carry HLA-DQ8. The negative predicting value of the two tests combined is 99%. However, 20-40% of the general population carries one or both of these haplotypes. Therefore, this test can only be used to exclude coeliac disease as a possible diagnosis. Especially in patients on a gluten-free diet, this can be beneficial. Tissue Transglutaminase IgA antibodies will not be detectable after a year-long strict gluten-free diet adherence.*

The patient carries the HLA-DQ2.5 haplotype; coeliac disease cannot be rejected as a diagnosis.

1. **Would you perform a gluten challenge on this patient ( the dietary re-introduction of gluten, followed by a serological antibody test)?**

- Yes
- No

*You decide to initiate a gluten-containing diet in this patient and test for anti-tTGA IgA after a while.*

1. **What should be the duration of the gluten exposure?**

…

_ The end of case 2 _

## Case 3

A 2,5-year-old toddler is presented by his mother because of faltering growth, excessive crying and a low food intake. His mother has biopsy-proven coeliac disease, which increases your suspicion of coeliac disease in the toddler. You order an anti-tTG IgA serology test.

| **Test** | **Result (normal value)** |
| --- | --- |
| anti-tTGA IgA | 92 U/ml (< 7 U/ mL) |
| IgA deficiency | No |

1. **Based on the test results, what would be your next step in the diagnostic process?**

- The initiation of a gluten-free diet
- Referral to a dietitian
- Referral to a gastroenterologist
- Referral to a paediatrician
- You order a gastroduodenoscopy with biopsies
- Other: …

*The paediatrician confirms the diagnosis of coeliac disease.*

1. **The toddler has two sisters, of five-year-old and seven-year-old. Both of them are healthy and are developing at an average pace. Will you test the sisters for coeliac disease?**

- No
- Yes
  - **If you’ve answered ‘yes’ in the previous question, what would be your first step in the process?**
    - A gastroduodenoscopy with duodenal biopsies
    - HLA-DQ2/8 typing
    - Anti-tTGA IgA antibody test

_ The end of case 4 _

## Case 4

A 28-year-old female visits your clinic. Her sister was diagnosed with coeliac disease by a gastroenterologist. Since she is a first-degree relative, your patient was also tested for coeliac disease by this gastroenterologist using an anti-tTGA IgA antibody test (which turned out positive), and the diagnosis was established based on a histopathological examination of a biopsy.

She now turns to you because she has no complaints whatsoever and wonders if a gluten-free diet is needed.

1. **What would be your advice?**

- Initiate a gluten-free diet
- Initiate a gluten-restricted diet
- No restriction of gluten whatsoever; in case of complaints she should return
- A check-up of Hb, ferritin, folate acid, vitamin B12, calcium, albumin, alkaline phosphatase, TSH, anti-tTGA IgA antibody every one or two years

_ The end of case 4 _
